# A Computational Screen for Type I Polyketide Synthases in Metagenomics Shotgun Data

**DOI:** 10.1371/journal.pone.0003515

**Published:** 2008-10-27

**Authors:** Konrad U. Foerstner, Tobias Doerks, Christopher J. Creevey, Anja Doerks, Peer Bork

**Affiliations:** European Molecular Biology Laboratory, Heidelberg, Germany; NERC Centre for Ecology and Hydrology, United Kingdom

## Abstract

**Background:**

Polyketides are a diverse group of biotechnologically important secondary metabolites that are produced by multi domain enzymes called polyketide synthases (PKS).

**Methodology/Principal Findings:**

We have estimated frequencies of type I PKS (PKS I) – a PKS subgroup – in natural environments by using Hidden-Markov-Models of eight domains to screen predicted proteins from six metagenomic shotgun data sets. As the complex PKS I have similarities to other multi-domain enzymes (like those for the fatty acid biosynthesis) we increased the reliability and resolution of the dataset by maximum-likelihood trees. The combined information of these trees was then used to discriminate true PKS I domains from evolutionary related but functionally different ones. We were able to identify numerous novel PKS I proteins, the highest density of which was found in Minnesota farm soil with 136 proteins out of 183,536 predicted genes. We also applied the protocol to UniRef database to improve the annotation of proteins with so far unknown function and identified some new instances of horizontal gene transfer.

**Conclusions/Significance:**

The screening approach proved powerful in identifying PKS I sequences in large sequence data sets and is applicable to many other protein families.

## Introduction

The majority of the microorganisms on earth cannot be cultured under standard laboratory conditions [1]. Therefore, uncultured organisms from environmental samples are promising sources of new enzymes and chemical compounds with biotechnological and pharmaceutical applications. Currently, three screening techniques are commonly applied for exploring protein functions in environmental samples: the function-based, the sequence-based and the substrate-induced gene-expression screening (SIGEX) [2]. Here we present a framework for sequence-based computational screens in environmental shotgun sequences, i.e. metagenomics data. It involves both homology-based and phylogenetic classification. While there has been some success in identifying important subfamilies in metagenomics data [3]–[6], there are also immense challenges ahead as tools and computational infrastructure often do not scale with the increase in metagenomics data and as many protein families have complicated evolutionary histories.

In order to explore a difficult and also important protein family in the context of diverse metagenomics data sets, we have chosen type I polyketide synthases (PKS I) as target proteins for screening. They synthesize a highly diverse group of secondary metabolites that covers many biological functions and have considerable medical relevance. Polyketides in general can act among other functions as antibiotics, immunosuppressants, pigments but also as toxins or carcinogens [7] via different mechanisms. Antibiotics like Erythromycin, Rifamycin and Oleandomycin are only a few examples with medical relevance. Polyketides are usually large chemical compounds that are synthesized in a series of repetitive steps. Similar to the synthesis of fatty acids short acyl-units are added to the growing molecule and are modified. All of these steps are catalyzed by a combination of domains, namely a acyltransferase domain (AT – transfers the acyl unit to the acyl carrier protein), a ketoacyl synthase domain (KS - performs the decarboxylative condensation), and an acyl carrier protein (PP - contains the phosphopantetheinyl arm) domain. Additionally the ketoreductase (KR), the dehydratase (DH), the enoyl reductase (ER) and the methyltransferase (MT) domain can modify the acyl unit after the condensation. The thioesterase domain (TE) releases the finished polyketide. PKS members have been found in bacteria, fungi, plants, slime mold [8], Alveolata [9] and animals [10], [11]. Like the fatty acid synthases (FAS), PKS are classified according to the arrangement of their domains: type I with multiple domains per protein and type II in which each single domain represents an independent protein. Bacterial type I PKS are usually modular where each module is responsible for a single fusion step [12] while fungal type I PKS proteins usually occur as “iteratively” acting enzymes in which the domain combinations catalyze several steps. In plants a third class - PKS type III (chalcone synthases) – was discovered and later also described in bacteria [13]. It is common to classify the PKS into these three types although many exceptions of this classification are known [14], [15] as the evolution of PKS is rather complex [10], [12], [16]–[18].

There have been numerous attempts to identify PKS in environmental samples using non-computational methods (e.g. [19]). Here, we present a computational approach based on Hidden-Markov-Model (HMM) sequence searches (as done in other PKS focused studies like [20]) followed by the construction of maximum-likelihood trees. This allows us to screen for multi-domain proteins and to estimate the potential of the different environments to serve as a source of PKS I sequences. Although the discrimination of type I PKS from type II PKS and type II FAS is simple, due to the large evolutionary distance [12] and PKS III are also a clearly separable group, a unique PKS I identification remains challenging. Reasons among others are the paralogy of type I PKS with type I fatty acid synthases [12] and with other enzymes and the fast evolution of PKS I. As PKS I proteins can be very large, it is unlikely that complete proteins are found in the highly fragmented shotgun metagenomic sequences. However, their multi-domain, repeated structure provides multiple instances of evidence to find real PKS I orthologs when searching independently with HMM of each of the eight domains introduced above.

Our approach included the creation and use of domain specific HMMs to find members of the type I PKS domain in six published metagenomic data sets - Minnesota farm soil (MSF) [21], Sargasso Sea (SGS) [22], human gut (HGUT) [23], acid mine drainage (AMD) [24], enhanced biological phosphorus removal sludges (EBPRS) [25] and whale falls (bones from sunken whales) (WLF) [21]. We used the UniRef database [26] as an reference set by treating it as another sample to be able to identify biases and the status of PKS I annotation. In contrast to most other studies that cover computational PKS analysis we did not only focus on AT and KS domains but took all eight domains into account. The results of the searches were the basis for the construction of maximum-likelihood trees which allowed the more precise classification of the HMM hits into type I PKS and non-PKS I members.

## Results

### Extracting PKS I candidate sequences using Hidden Markov Models

From 926 annotated type I PKS domain sequences in the PKSDB dataset [27], we generated multiple alignments and constructed eight Hidden Markov Models (one for each domain) that were searched against 6,613,204 predicted proteins in six metagenomics samples and UniRef (for details see methods).

In total 22,106 candidate sequences of the eight PKS I domains were retrieved and analyzed. They range from 45 MT domain sequences to 4355 sequences of the KS domain type (for individual datasets see Table S1). For most of the domains the UniRef set has the highest total and relative (compared to the total number of analyzed proteins) number of candidate type I PKS.

### Refining potential PKS I sequences using maximum likelihood trees

Although we did not find type II PKS sequences, due to the similarity of PKS I to FAS I and other enzymes, HMMs alone were not sufficient to discriminate PKS I proteins and related enzymes. Therefore, we applied a phylogenetic approach [28] which allowed the subsequent characterization of type I PKS subgroups.

**Figure 1:**
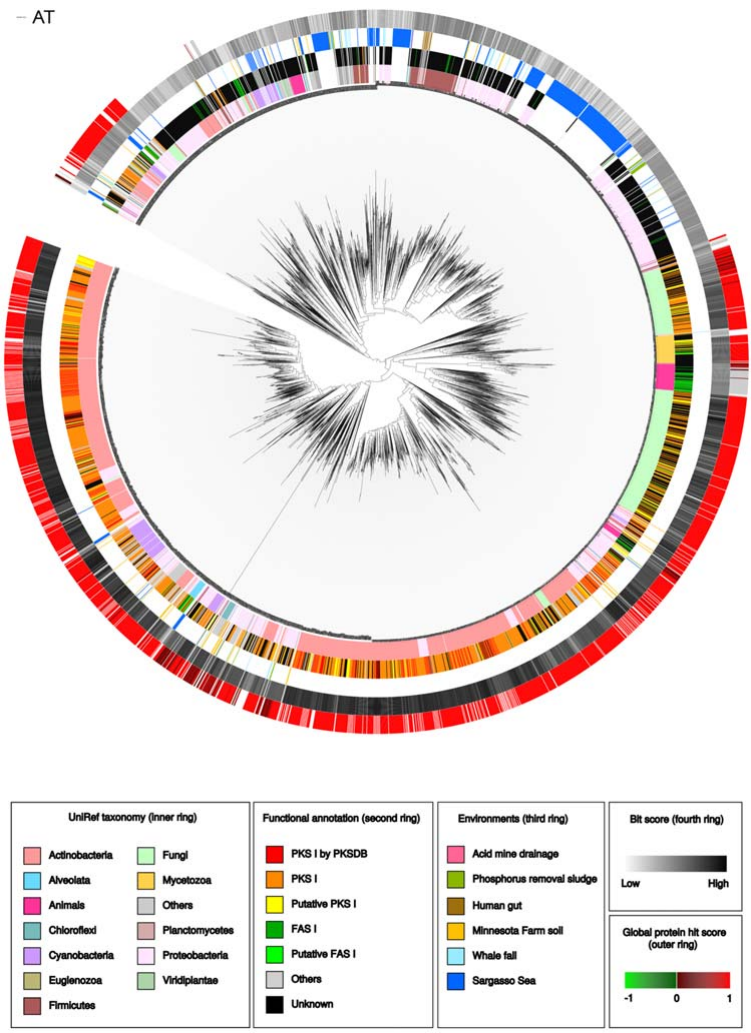
Maximum likelihood-tree of the AT domain.

In agreement with previous knowledge the trees of the AT, DH, ER, KR, KS and PP domains show in general a consistent phylogenetic profile and contain PKS I and non-PKS I taxa (see Fig. 1 as an example, all other trees can be found in Methods S1 and S2). The main fraction of leaves in the PKS I branches is contributed by the Actinobacteria and clusters mostly together (see Table S2). Members of the Proteobacteria and other bacteria phyla occur in mixed groups. The fungal sequences form in most of the trees one or two groups within the PKS I branch and are closely located to sequences of other eukaryotes like *Dictyostelium* and animals. It was previously described that most of these animal proteins are FAS I members which are phylogenetically related to the fungal type I PKS [12], [16] and also the occurrence of PKS-like sequences in animal genomes (e.g. in sea urchin for the production of pigments) has been reported [10].

Not all domains perform equally in identifying PKS I members. For example, in the TE domain tree two clades are dominated by PKS I sequences but a clear discrimination between PKS I and non-PKS I members cannot be made for the rest of the tree. For example, the MT domain tree contains only a few members as the domain occurs quite rarely in type PKS I; also due to the short length of the PP domain the results in this tree are less resolved than those of the other seven domains.

The non-PKS I branches are large in some trees. In particular, in AT, KS and TE domain trees many unspecified acyltransferases, ketoacyl synthases and thioesterases respectively, were apparently not filtered out by the HMM searches. In the DH domain tree the non-PKS I sequences are predominately annotated as FAS members while ER and KR domain HMM searches seems to attract non-specified dehydrogenases and other oxidoreductases. The non-PKS I PP domain members were mainly adenylate amino acids or nonribosomal peptide synthetases (NRPS).

### Quality analysis of the tree-based approach and HMM searches

The enormous computational requirements of the tree reconstructions made bootstrap analyses infeasible. However, the fragmented environmental sequences could strongly influence the quality and significance of the branches. We thus compared the trees with reference trees without metagenomic sequences and randomly created trees with the same amount of taxa. The Robinson-Foulds distances [29] between the test trees and the references trees were in general much smaller than the distances to random trees (see Fig. S2, Table S3 and Methods S3). Also, the log likelihood of the reference tress and trees with metagenomics samples show a much better fit to the sequence alignments and are much more similar to each other than to trees with random topologies (see Methods S3 and S4). This implies that the trees are a good representation of the phylogenetic signal in the dataset and that their topologies are not overly influenced by the inclusion of the metagenomic sequences.

To support the tree-based annotation of the metagenomics sequences, the placements of all manually annotated PKS I from PKSDB were checked. They should only be found in branches of the trees that are marked as PKS I containing branches. With exception of the TE domain set which has three PKSDB sequences that are located in non-PKS I branches (see Methods S5) all sequences are placed as expected in PKS I branches.

**Figure 2:**
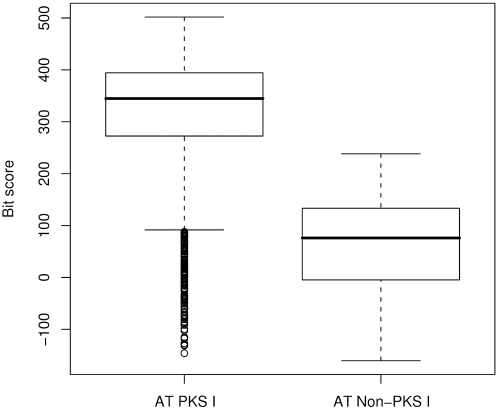
Box plots of the bit score distribution of HMM search result sequences for the AT domain classified as PKS I or as non-PKS I using the tree.

Using the trees for classification, it became apparent that the HMM bit score values are not a sufficient criterion for discriminating the type I PKS from the non-PKS I sequences. To quantify this, sequences of the HMM searches were grouped by their tree based annotation (implying that this is close to the true function). The bit score distributions of these groups were compared domain-wise and plotted as box plot (Fig. 2 for the AT domain, Fig. S1 for all domains). All domains have a higher median value for the PKS I than the non-PKS I. But for most of the domains there is a large overlap of the bit score value between these groups. Especially the many outliers with low bit scores in the type I PKS group coming from metagenomic proteins fall in the inter-quartile range of the non-PKS I group.

Taken together, these quality measurements indicate that the tree approach can properly classify the candidate sequences retrieved by HMMs into PKS and non-PKS I members.

### PKS I domain densities in various environments

**Figure 3:**
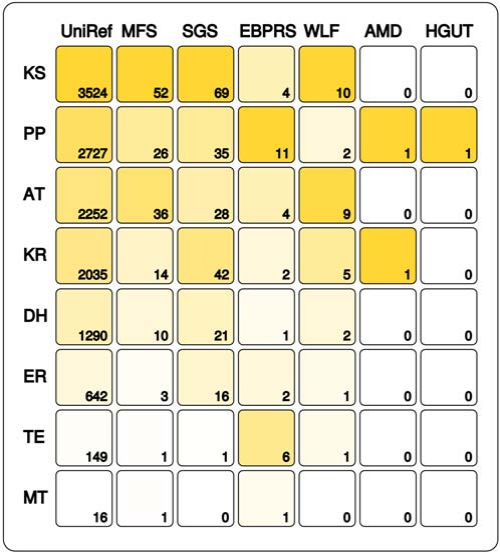
Number of sequences in the data sets that are annotated as type I PKS domains based on the maximum-likelihood tree. The intensity of the color is equivalent to the relative number of sequences inside a data set. The KS domain has in the larger data sets the highest number of hits and the ratio of the AT, KS and PP domain is mostly similar.

The number of domains that fall in branches which are classified as type I PKS members as they contain known PKS I sequences are visualized in Fig 3. In nearly all seven data sets the KS domain is found most frequently (with the exception of enhanced biological phosphorus removal sludge data sets) followed by the AT, PP or KR domains. ER and TE sequences occur generally in much lower counts. In agreement with previous studies the MT domain appears very rarely and could only be found in UniRef, the Minnesota farm soil sample and the phosphorus removal sludge. The discrepancy between the AT and KS domain occurrences might indicate different, domain specific HMM sensitivities as they tend to occur at equal copies, but it could have also biological reasons as the number of AT domains in PKS I proteins might differ from the number of KS domains if a trans-acting AT domain is involved [30].

**Figure 4:**
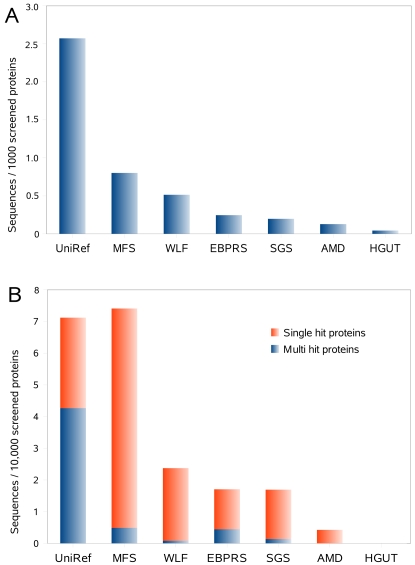
A – PKS I classified sequences normalized by total number of screened proteins. B – PKS I classified sequences normalized proteins-wise (all domains of one protein are counted together as one entity) by total number of screened proteins.

The density of PKS I domains has the highest value in UniRef when the number of tree-refined PKS I sequences is normalized by the total number of proteins in each of the data sets (Fig 4A). It is around three times higher than that of Minnesota farm soil sample which has the highest in all environments.

In UniRef, many different PKS I domains are found in the same protein while the metagenomic sequences mostly encode protein fragments with a single domain due to the shotgun approach taken during data generation. Assuming that each of these metagenomic domain sequences represent a full type I PKS protein we normalized the number of single and multi domain hit proteins by the number of screened proteins (Fig. 4B). We found that only the farm soil has a higher PKS I density than UniRef, and PKS I seem most rare in the gut sample where only a single domain occurrence could be detected.

**Figure 5:**
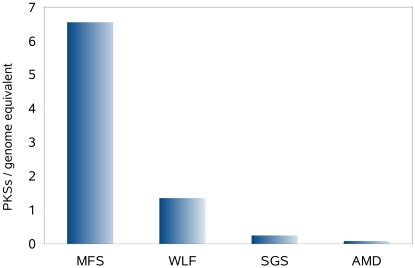
Type I PKS members per genome equivalent for the Minnesota farm soil, whale falls, Sargasso Sea and acid mine drainage sample estimated by Raes et al. [31]. The soil sample has the highest density of type I PKS per genome.

The identified PKS I proteins were also normalized by the number of genome equivalents for the Minnesota farm soil, Sargasso Sea, whale falls and acid drainage mine data sets as for these environments average effective genome sizes have been estimated [31]. With nearly seven type I PKS per genome equivalent, the farm soil has the highest density of these proteins (Fig. 5). This is in the range of fully sequenced genomes of organisms from soil habitats [12].

In UniRef, the largest proportion of potential PKS I proteins identified originated from Actinobacteria (5642 sequences), followed by Proteobacteria (3625 sequences). This is similar to statements of previous studies and may be biased by the number of sequenced genomes of these phylogenetic groups [12]. The counting of all taxonomic groups can be found in Table S2. We did not find potential type I PKS members in archaeal proteins. A possible reason for this is the lack of an FAS AT domain in archaea [12] and the low likelihood of horizontal transfer of PKS I genes. As the source organisms of proteins from environmental samples are unknown, a detailed analysis of the taxonomic distribution is currently impossible.

As expected, the majority of the environmental sequences are located in clades dominated by bacterial PKS I domains, but there are metagenomic sequences that seem to have a closer relationship to eukaryotic type I PKS members. For example six Sargasso Sea sequences can be found close to *C. elegans* and Alveolata proteins in the AT domain tree. The originating species of these sequences is unfortunately unclear.

Despite the fragmentation of the metagenomic sequences we were able to find proteins with multiple domains in some of the six environments. In the Sargasso sea sample, 15 of these with a maximum number of seven domains were detected. The farm soil collection hosted nine multidomain proteins but none extended beyond two domains. The phosphorus removal sludge set contained six (up to three domains) and the whale fall one (two domains) of such sequences. The small number of multi domain hits found reflects the low coverage of the samples. But the fact that at least some are found give high confidence that we have detected real PKS I members and that these communities might be useful as sourced for further and more focused sequencing and screenings.

### Distribution of potential type I PKS members in the different Sargasso Sea samples

The Sargasso Sea data set is composed of seven samples. It has been suggested that sample 1 of the Sargasso Sea data set was contaminated with *Burkholderia* and *Shewanella* species [32]. To exclude the possibility that this contamination biased the identification of PKS I proteins, the sample of origin of each of protein identified was examined. Additionally, their closest relatives in UniRef were determined by using BLAST. We found that seven of the 15 proteins with multiple domain hits were encoded by contigs mainly built from sample 1 reads, four from *Burkholderia* and two from *Shewanella*. Of the 171 single-domain hit proteins in the seven Sargasso samples, only 27 are found in contigs with contributions of sample 1 and none of these seems to be close related to *Burkholderia* proteins or *Shewanella* proteins. The high number of multi-domain protein hits coming from potential contaminations may be a result of the better coverage of these genomes in the first sample. However, the remaining single-domain hit proteins provide enough evidence that type I PKS proteins are not solely due to the contaminating species but that the uncontaminated ocean sample also hosts type I PKS producing organisms.

### Detection of non-annotated PKS I members in UniRef

The screening and tree based refinement of UniRef proteins revealed type I PKS members that were so far not annotated as PKS I or PKS at all. This includes 971 proteins with multiple PKS I domain HMM hits and 760 proteins (mostly short, fragmented ones) with only one such hit. Additionally we could confirm the proposed annotation of further proteins, 197 proteins with multiple domain hits and 146 proteins with single domain hits, that were marked as hypothetical, putative, probable or predicted PKS or PKS I.

The classification and functionality of PKS proteins in animals is still unclear. Based on the analysis of AT and KS domains Jenke-Kodama et al. [12] placed the animal FAS into the type I PKS family which makes them a subfamily of PKS. Castoe et al. [10] showed that sea urchins (*Strongylocentrotus purpuratus* and *Lytechinus variegatus*), birds (*Gallus gallus*), and fish (*Danio rerio* and *Tetraodon nigroviridis*) harbour PKS-like proteins with uncertain functionality, which are closely related to PKS members of *Dictyostelium*. In our study, the Metazoa contributed proteins with AT and KS domains (in some cases also the ER domain) that were placed in the PKS I branches of the trees while the remaining domains were found in non-PKS I branches. This distribution was the case for some insects, amphibia fish, echinodermata and mammals. In contrast all detected six domains of a protein in *Caenorhabditis briggsae* and eleven domains (except one DH domain) in *Caenorhabditis elegans* seem to be true type I PKS domains.

The proteins in the Alveolata *Cryptosporidium hominis*, *Cryptosporidium parvum*, *Toxoplasma gondii* are very large and contain only PKS I annotated domains. It confirms the described occurrence of PKS I in the protozoan pathogen *Cryptosporidium parvum* [9]. The detection of type I PKS members in *Ostreococcus tauri* and *Ostreococcus lucimarinus* sequences in UniRef supports a study that reported type I PKS proteins in unicellular green algae based on a KS domain tree [33]. The PKS I of these protists are described to be different from the currently known PKS proteins and might have a long separated evolution. The different domains detected were found to be placed close to disparate taxonomic groups (within bacteria and eukaryotes) in the trees generated.

### Indication of horizontal gene transfer

The constructed phylogenetic trees also revealed some cases of potential horizontal gene transfers. An example is a small group of 3 fungal protein taxa in the AT domain tree that is placed in the Actinobacteria. In the DH domain tree, four *Danio rerio* (zebra fish) sequences are nested in a small group of fungal sequences that is surrounded by sequences from Actinobacteria. All proteins have the same domain structure including a KS, AT and KR domain in addition to the DH domain. It cannot be excluded that the detected protein originated from a genome contamination though. Protein identifiers of the described cases are listed in the Metods S3.

## Discussion

Because of their size, modular structure, complicated evolution and similarity to type I FAS and other enzymes, PKS members are a challenging group of enzymes to identify and to classify. We were able to detect type I PKS proteins – one subgroup of the PKS group - in almost all the samples studied (Fig. 3). The Minnesota farm soil sample shows the highest density of PKS I which is not surprising as this environment has the highest species density which leads to strong competition and an “arms race” between species. The enormous potential for soil as source of useful secondary metabolites was already discussed earlier [34] and our results support these statements.

For both the human gut (145 Mb of reads, 46503 predicted genes) and acid mine drainage samples (140 Mb, 46862 predicted genes), the HMM searches identified only one candidate PKS I, albeit with high similarity to known PKS I sequences. This implies a low PKS I density in these environments and it has to be proven whether the respective species are members of the microbial communities or just temporal bystanders that came in via food or air. At least for AMD, one of the two detected PKS I proteins was found in one of the major community members, the *Leptospirillum* group III. This implies that even in an inhospitable environment like AMD, which contains only a small number of species, the community forces its inhabitants to arm themselves with expensive secondary metabolites. These kinds of environments have so far not been considered as sources of PKS proteins but our study indicates that novel attempts to search for antibiotics and other metabolites in them may reap rich rewards.

In addition to a quantification of PKS I in diverse environments, our study has also helped to classify unknown proteins in UniRef and improved their annotation. The usage of phylogenetic trees to discriminate between PKS I and non-PKS I sequences seems to be a feasible approach which also partially overcomes the problem of low bit score values and fragmentation of environmental proteins using traditional sequence similarity searches. Depending on the target sequences this method can be successfully applied to search in Sanger sequencing data sets and new generation 454 pyrosequencing data sets with read lengths starting from 450 bp (see Methods S4). The approach also shows the limits of current annotation schemes: If HMM searches had been the only approach used, this would have resulted in many false positives and false negative PKS I being identified. Despite this, the HMMs used here have been carefully designed, appear PKS I specific and are much more discriminative than those currently available (e.g. in PFAM [35] or TIGRFAM [36]). The HMMs have been deposited in SMART [37]. The combination of the information of all eight domain searches was shown to be a powerful detection method.

The approach outlined here can be applied to search further proteins of interest in environmental shotgun sequences and has been already successfully used to screen for the much smaller family of Nitrilases [6]. The rapidly increasing amount of metagenomic data that will be publicly released requires methods such as the one presented here to quickly and cheaply screen for proteins of interest.

## Materials and Methods

### Metagenomic and reference data sets

Sets of predicted proteins from the following metagenomics samples were analyzed in this study: Minnesota farm soil [21], Sargasso Sea [22], human gut [23], acid mine drainage [24], enhanced biological phosphorus removal sludges [25] and whale falls (sunken whale bones) [21]. Additional to the metagenomic samples proteins sequences from UniRef100 database [26] were used as reference set.

### Hidden-Markov-Model creation and search

Due to the fact that neither Pfam [35] nor other resources offer Hidden-Markov-Models (HMM) of all the the eight PKS I domains, they were constructed based on a manually curated set of PKS I protein sequence hosted at PKSDB [27]. For each domain the sequences were aligned with *muscle* [38]. Based on these alignments HMMs were created and calibrated by *hmmbuild* and *hmmcalibrate* HMMER-package [39]. The UniRef protein sequences were screened with these HMMs. Alignments (by *muscle*) of extracted proteins were used to calculate maximum likelihood trees. The trees helped to manually select real PKS I members that were afterward aligned again. After a manual cleaning of these alignments they were used to generated HMMs (with the above described tools). Searches for type I PKS domains in the metagenomic sequences and UniRef were performed with these PKS I domain specific HMMs. A non-HMM based searching approach can be found in Methods S4 and Table S4, S5 and S6.

### Tree construction

For each domain the sequences detected by the HMM were filtered by their e-values (see Methods S4). The selected sequences from UniRef and the metagenomic datasets were aligned by *hmmalign* (included in the HMMER-package [39]). For the KS and PP domain the UniRef sequence collection was shrunk to a set of representatives by making use of *blastclust* (from the NCBI BLAST package [40]) and a Python script [http:python.org]: Clusters based on a similarity cut-off of 90% were created and the annotation strings checked if all members were either PKS I or non-PKS I sequences. Without the resizing these two datasets would have been too large for further processing by *phyml*. Based on the alignments maximum likelihood trees were constructed using a slightly modified (removing limitation for memory usage - see Methods S6 for the patch file) version of *phyml* [28].

### Data base construction and querying

Information like fasta file headers, HMM result quality, tree position and manual, tree based classification of the sequences were combined in a *sqlite* database [http://www.sqlite.org] that was queried to created result statistics (see Methods S5).

### Comparison of the tree topologies with reference trees

To test if the noise from the fragmented metagenomic samples overwhelms the phylogenetic signal of the reference set sequence from UniRef, a reference tree based on the alignments for the HMMs was built for each domain. The tree containing the environmental sequences and the reference trees were then pruned to their set of common taxa using *clann* [41]. For each domain 500 random trees containing the same leaf set as these common taxa trees were generated by the program *random_tree* (see sumplementary material) using a markovian approach. The pairwise Robinson-Foulds distances [29] of all combinations of these 502 trees were calculated with the *rfdist* function of *clann*. Supported by a python script box plots were created using *R* (http://www.r-project.org/).

### Visualization and manual annotation

We used iTOL [42] for manual rerooting and visualizing of the trees. Tree nodes of proteins derived from UniRef or PKSDB were colorized by the taxonomic classification of the hosting species (different levels based on NCBI Taxonomy [43]). In addition automated, keyword based analysis of the annotation strings lead to a second color ring of the UniRef taxa. Further a source classifying color code was applied to environmental protein nodes. Both, UniRef and environmental proteins were marked by a color ring that reflects a value that we dubbed “global protein hit score” (GPHS). It is the difference of the number of domains in protein that are placed in PKS I branches and number of domains that are place in non-PKS I branches, divides by the total number of found domains (n_PKS_−n_Non_PKS_/n_PKS_+n_Non_PKS_). Proteins with a GPHS higher than 0 are more likely to be PKS, Proteins with a GPHS lower than 0 are more likely to be non-PKS I. The GPHS can only be calculated for multi domain hit protein.

For a visualization of the results, a program is provided that creates graphical overviews of the proteins and the detected domains based on the database content.

### Code and data availability

All python and C programs (Methods S6) that were created for this study are open source and available under the ISC license (http://www.opensource.org/licenses/isc-license.txt). The data base files and all other files are free availability under the Creative Commons Attribution License (http://creativecommons.org/licenses/by/3.0/).

The generated detailed results are available in the supplementary material. This includes the resulting sequences of the HMM searches (Methods S7), the alignments (Methods S7), the trees in Newick format (Methods S7), visualization of the trees (Methods S1 and S2) as well as the database that hold the integrated data (Methods S5). Also a text file of selected parts of the database is included (Methods S5). The created Hidden-Markov-Models are incorporate into domain search web service SMART [37].

## Supporting Information

Methods S1 Maximum likelihood trees of the AT, DH, ER, and KR domains
(7.24 MB ZIP)

Methods S2 Maximum likelihood trees of the KS, PP, MT and TE domains
(4.38 MB ZIP)

Methods S3 Box plots of bit score and Robison-Foulds distances distributions
(0.04 MB ZIP)

Methods S4 Supplementary Information
(0.46 MB PDF)

Methods S5 SQLite data base of the integrated information and tables of selected columns in CSV-format.
(4.39 MB ZIP)

Methods S6 Source code of programs (C and Python) and patches.
(0.06 MB ZIP)

Methods S7 Sequences, alignment and tree files of the different domains.
(6.92 MB ZIP)

Figure S1 Bit score distributions of the hits of HMM searches for all eight domains.
(9.61 MB TIF)

Figure S2 Robison-Foulds distances distributions
(2.88 MB TIF)

Table S1 Number of HMM search result sequences of different annotation classes
(0.11 MB DOC)

Table S2 Domain sequences marked as PKS I per taxonomic group
(0.10 MB DOC)

Table S3 Number of common taxa and Robison-Fould distances of the test and reference tress
(0.10 MB DOC)

Table S4 BLAST hits per domain in UniRef
(0.10 MB DOC)

Table S5 Counting of the group members of the multi hit proteins
(0.10 MB DOC)

Table S6 Counting of the group members of the single hit proteins
(0.10 MB DOC)
