# Supplementary material for: A Computational Screen for Type I Polyketide Synthases in Metagenomics Shotgun Data
Source: PLoS One. 2008 Oct 27;3(10):e3515. doi: 10.1371/journal.pone.0003515 (PMC2568958; doi:10.1371/journal.pone.0003515)
Supplement: Methods S1 — Maximum likelihood trees of the AT, DH, ER, and KR domains (7.24 MB ZIP) [file pone.0003515.s001.zip › trees_AT_DH_ER_KR/ER.pdf]

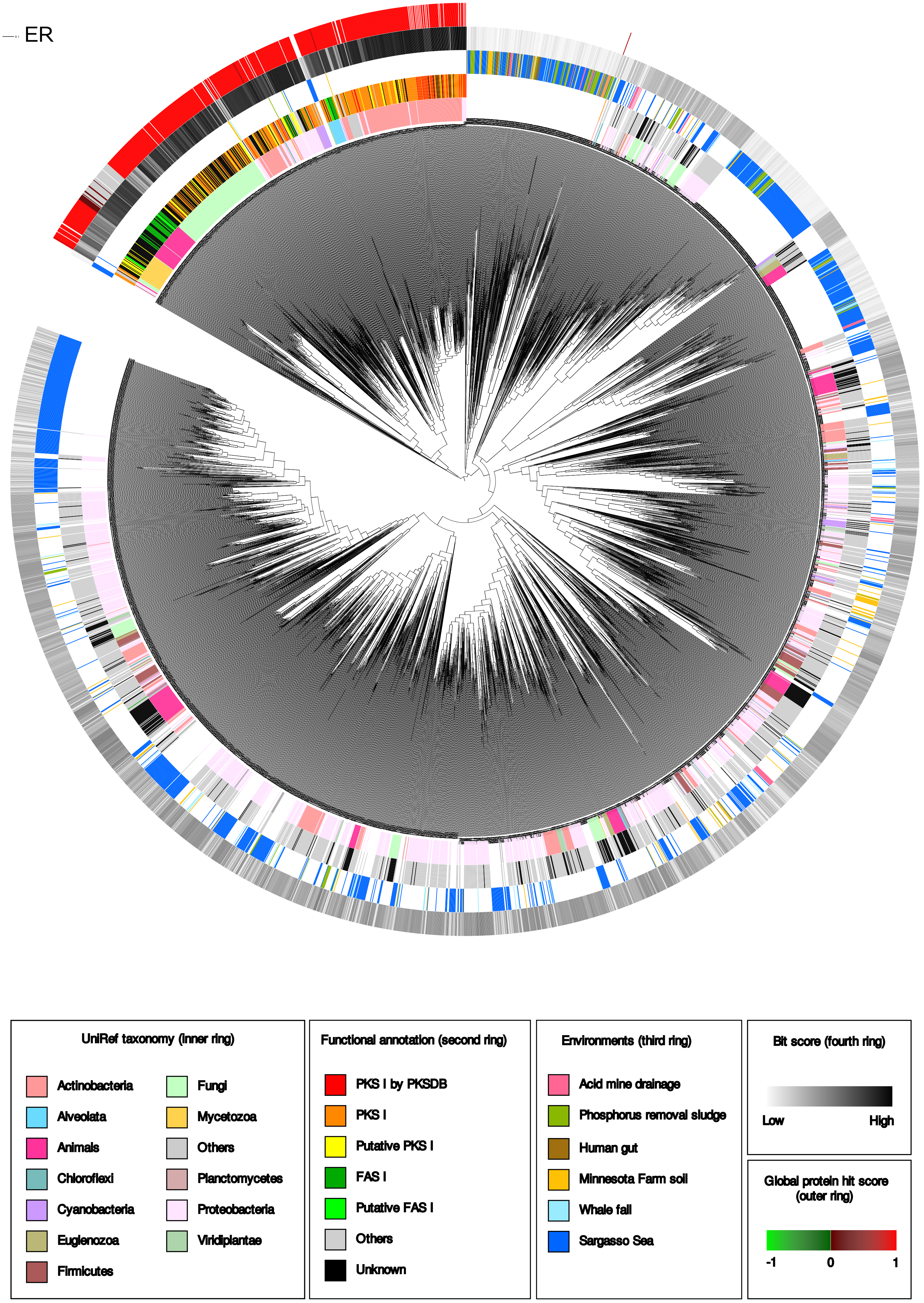
ER
UniRef taxonomy (inner ring)
Actinobacteria
Alveolata
Animals
Chloroflexi
Cyanobacteria
Euglenozoa
Firmicutes
Fungi
Mycetozoa
Others
Planctomycetes
Proteobacteria
Viridiplantae
Functional annotation (second ring)
PKS I by PKSDB
PKS I
Putative PKS I
FAS I
Putative FAS I
Others
Unknown
Environments (third ring)
Acid mine drainage
Phosphorus removal sludge
Human gut
Minnesota Farm soil
Whale fall
Sargasso Sea
Bit score (fourth ring)
Low
High
Global protein hit score (outer ring)
-1
0
1
