# Supplementary figures and images for: A Computational Screen for Type I Polyketide Synthases in Metagenomics Shotgun Data
Source: PLoS One. 2008 Oct 27;3(10):e3515. doi: 10.1371/journal.pone.0003515 (PMC2568958; doi:10.1371/journal.pone.0003515)

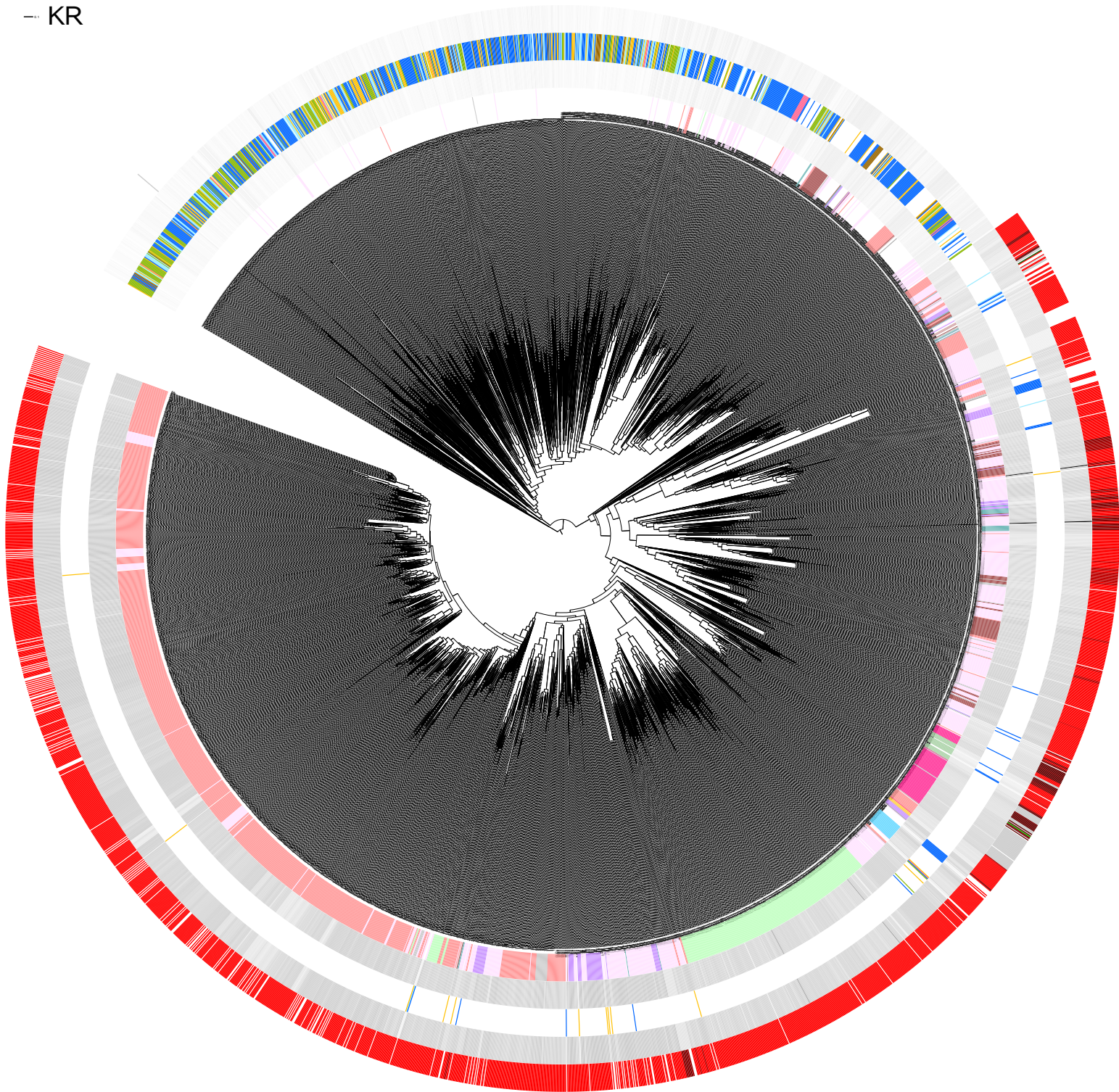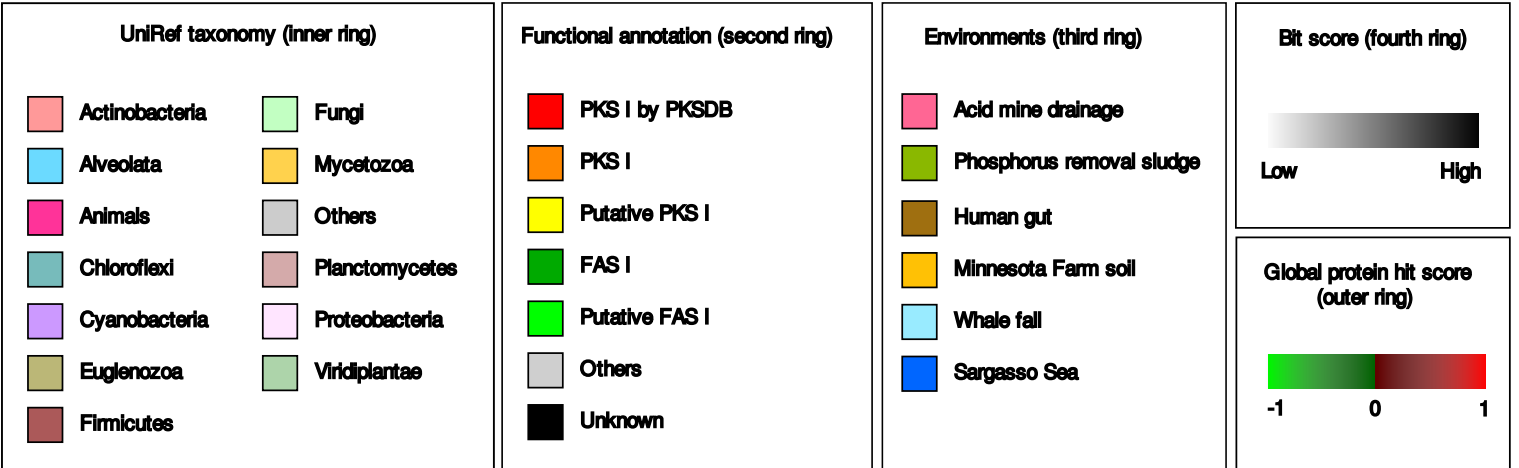

Supplement: Methods S1 — Maximum likelihood trees of the AT, DH, ER, and KR domains (7.24 MB ZIP) [file pone.0003515.s001.zip › trees_AT_DH_ER_KR/KR.pdf]

Bit score distributions

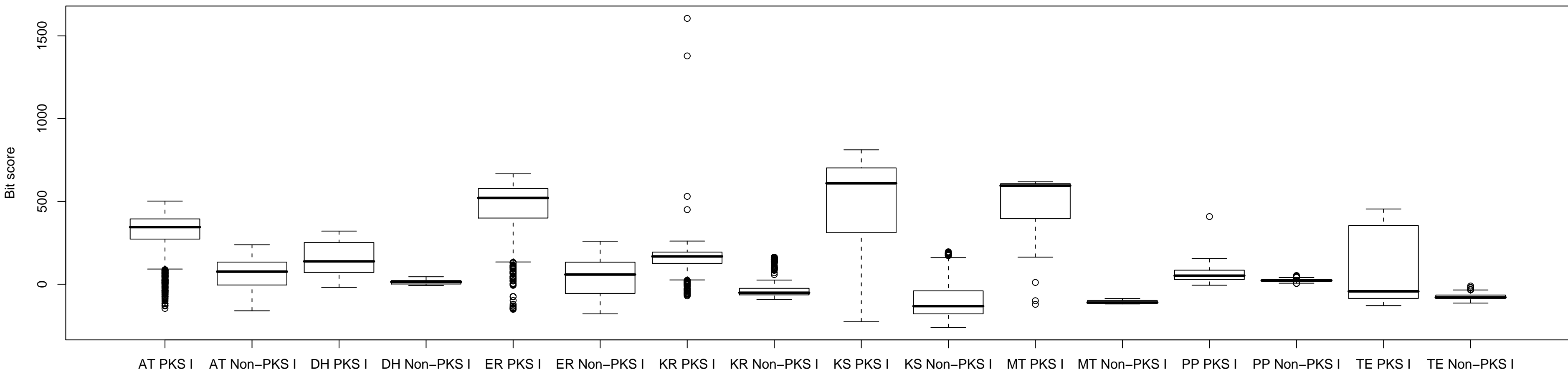

Supplement: Methods S3 — Box plots of bit score and Robison-Foulds distances distributions (0.04 MB ZIP) [file pone.0003515.s003.zip › plots/Sup_Fig-Bit_score_distritributions.pdf]

**1 reference tree + 1 full tree + 100 random trees  
= distribution of 5151 distances / per domain**

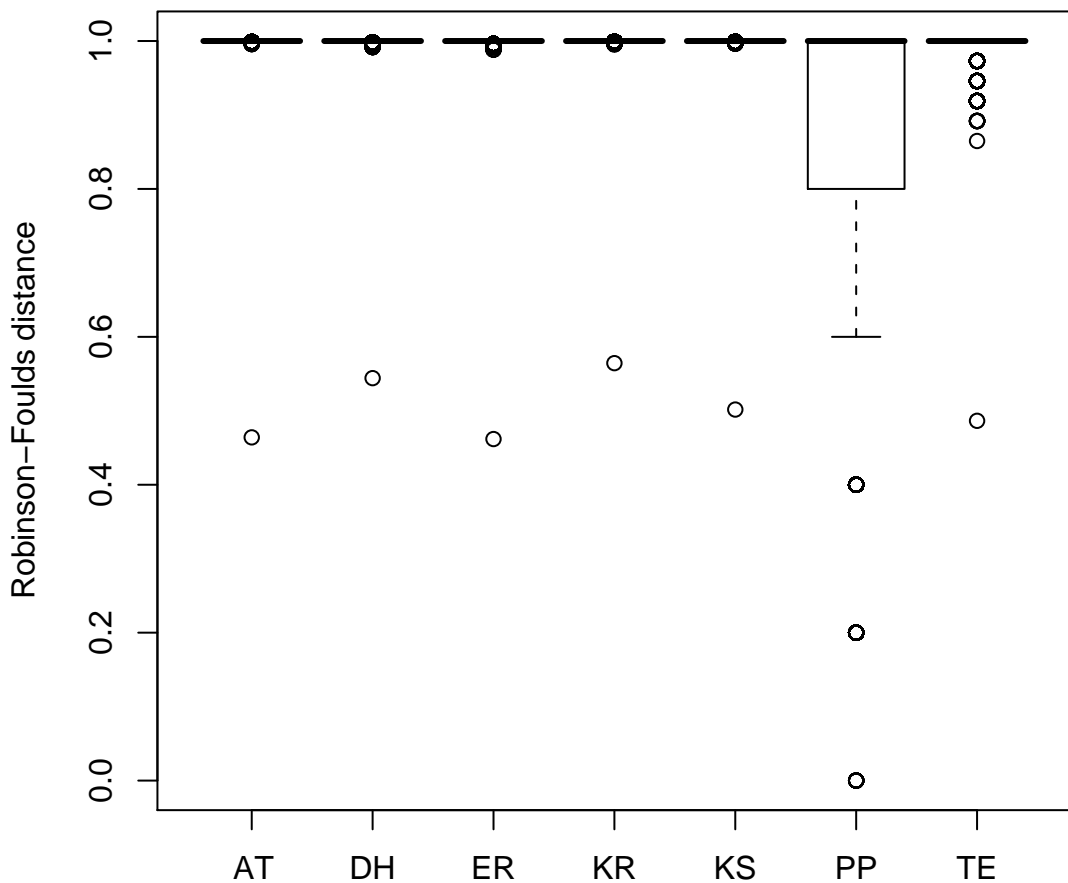

Supplement: Methods S3 — Box plots of bit score and Robison-Foulds distances distributions (0.04 MB ZIP) [file pone.0003515.s003.zip › plots/Sup_Fig-Robison-Foulds_distances.pdf]

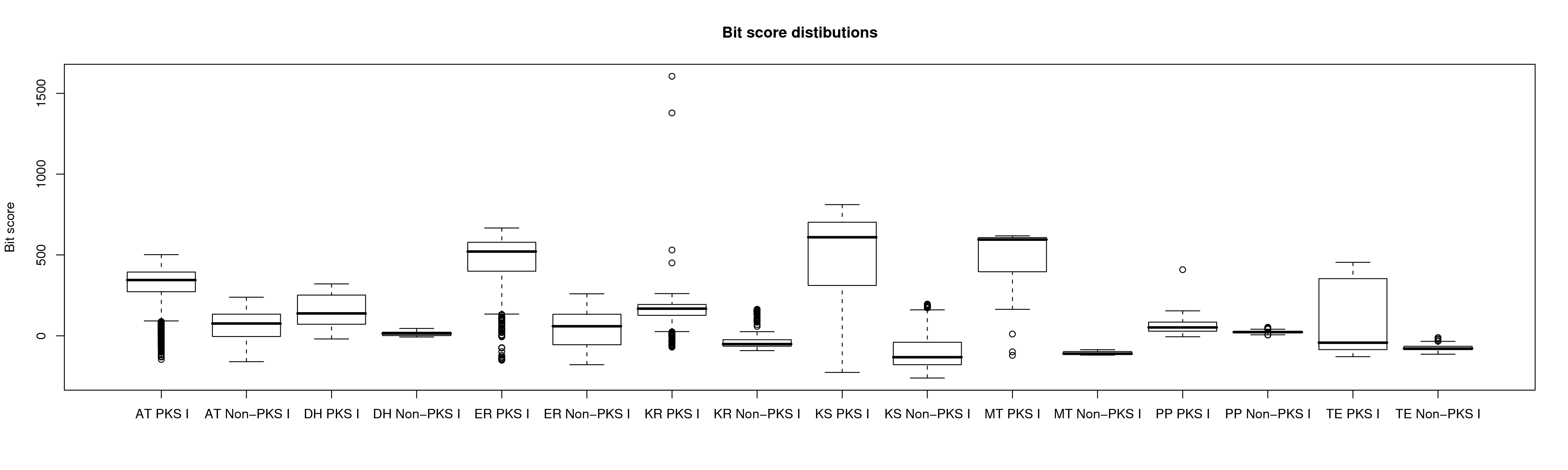

Supplement: Figure S1 — Bit score distributions of the hits of HMM searches for all eight domains. (9.61 MB TIF) [file pone.0003515.s008.tif]

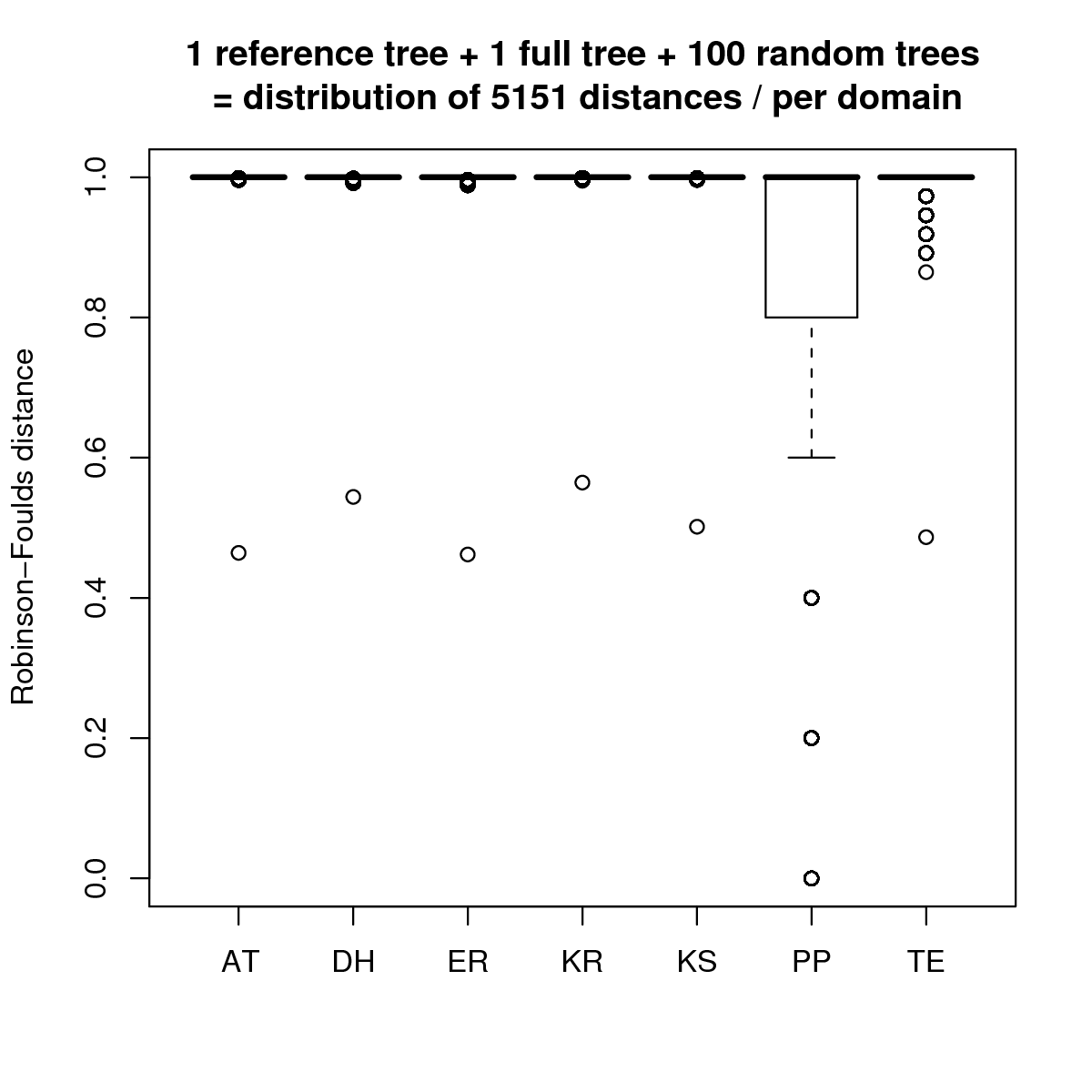

Supplement: Figure S2 — Robison-Foulds distances distributions (2.88 MB TIF) [file pone.0003515.s009.tif]
