# Supplementary material for: A Computational Screen for Type I Polyketide Synthases in Metagenomics Shotgun Data
Source: PLoS One. 2008 Oct 27;3(10):e3515. doi: 10.1371/journal.pone.0003515 (PMC2568958; doi:10.1371/journal.pone.0003515)
Supplement: Methods S2 — Maximum likelihood trees of the KS, PP, MT and TE domains (4.38 MB ZIP) [file pone.0003515.s002.zip › trees_KS_PP_MT_TE/MT.pdf]

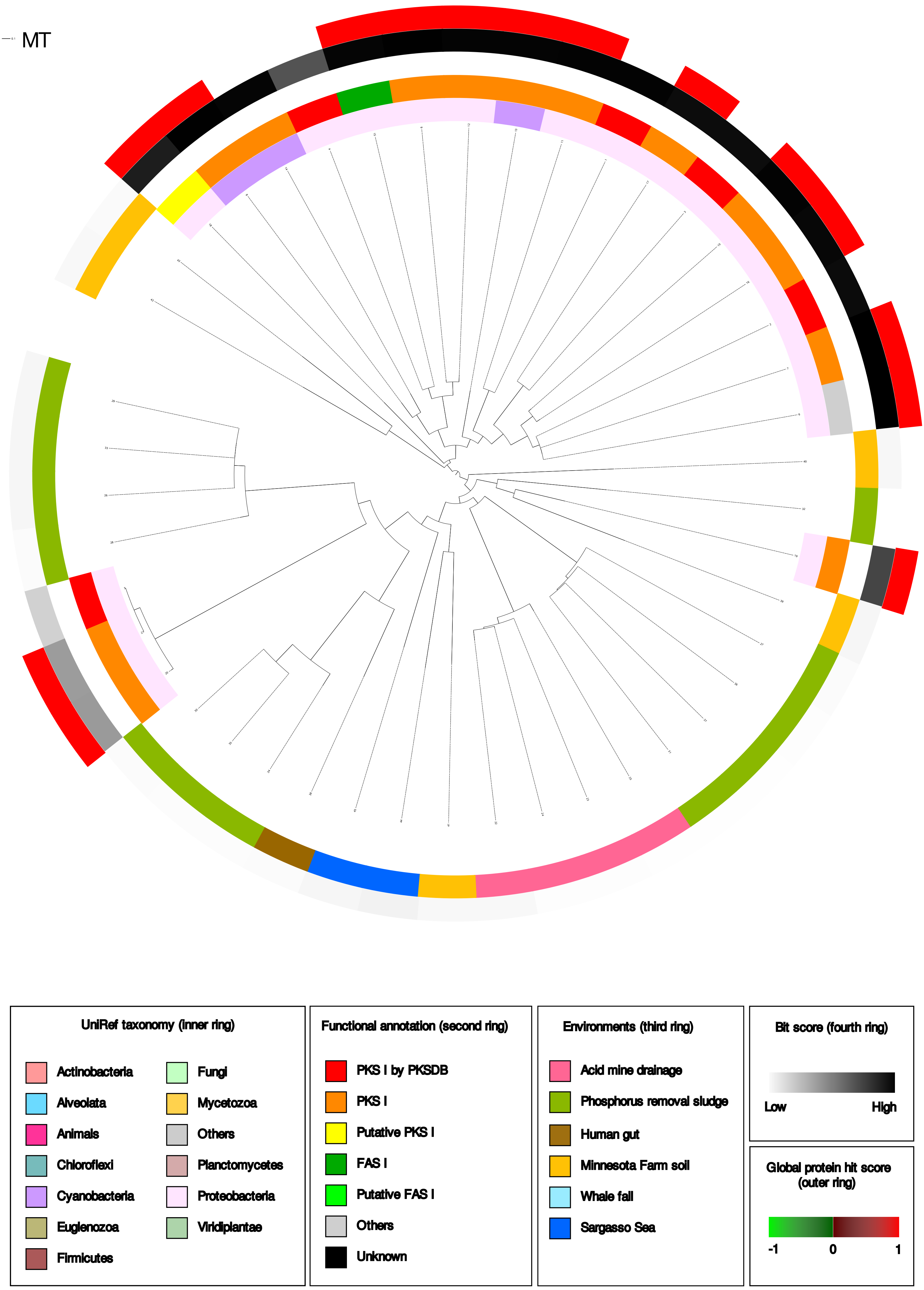
MT
UniRef taxonomy (inner ring)
Actinobacteria
Fungi
Alveolata
Mycetozoa
Animals
Others
Chloroflexi
Planctomycetes
Cyanobacteria
Proteobacteria
Euglenozoa
Viridiplantae
Firmicutes
Functional annotation (second ring)
PKS I by PKSDB
PKS I
Putative PKS I
FAS I
Putative FAS I
Others
Unknown
Environments (third ring)
Acid mine drainage
Phosphorus removal sludge
Human gut
Minnesota Farm soil
Whale fall
Sargasso Sea
Bit score (fourth ring)
Low
High
Global protein hit score (outer ring)
-1
0
1
