# Supplementary material for: A Computational Screen for Type I Polyketide Synthases in Metagenomics Shotgun Data
Source: PLoS One. 2008 Oct 27;3(10):e3515. doi: 10.1371/journal.pone.0003515 (PMC2568958; doi:10.1371/journal.pone.0003515)
Supplement: Methods S4 — Supplementary Information (0.46 MB PDF) [file pone.0003515.s004.pdf]

Supplementary information - A computational screen for type I polyketide synthases in metagenomics shotgun data

Konrad U. Foerstner, Tobias Doerks, Christopher J. Creevey, Anja Doerks, Peer Bork

European Molecular Biology Laboratory, Heidelberg, Germany

Hidden-Markov-Modell creation and search

The following e-values were used as cut-offs for the HMM searches:

- AT: 0.00001
- DH: 0.1
- ER: 0.0000000001
- KR: 0.00001
- KS: 0.000000000000001
- PP: 0.1
- TE: 0.1

Availability of the HMMs

The HMMs were included into SMART [PM 16381859] as named "PKS_AT", "PKS_DH", "PKS_ER", "PKS_KR", "PKS_KS", "PKS_MT", "PKS_PP", "PKS_TE").

Tree construction

In the alignment of the KS domain sequence UniRef100_A0ACI1_from_2372_to_3635 was manually removed as it increased the size of the alignment strongly.

Number of taxa in the trees

- AT: 3778
- DH: 1475
- ER: 4025
- KR: 3489
- KS: 3766 (+866 UniRef sequences filter out via *blastclust* = 4632)
- MT: 45

- PP: 3158 (+968 UniRef sequences filter out via *blastclust* = 4126)
- TE: 536

Tree evaluation

See Table S1.

Examples for horizontal gene transfer

The following HMM search result sequences are examples of a possible gene transfer, as they are fungal proteins (coming from *Aspergillus niger*, *Chaetomium globosum* and *Cochliobolus heterostrophus*) but occur with sequences from Actinobacteria in the AT domain tree:

- UniRef100_A2R3M8_from_1177_to_1493
- UniRef100_Q6RKE1_from_1178_to_1479
- UniRef100_Q2GZD3_from_1015_to_1323

The four *Danio rerio* DH sequences that are nested in a small group of fungal sequences that is surrounded by sequences from Actinobacteria:

- UniRef100_UPI0000F1DD85_from_895_to_1053
- UniRef100_UPI0000D8C28A_from_902_to_1060
- UniRef100_Q1MT73_from_895_to_1053
- UniRef100_Q5RJ12_from_902_to_1060

List of newly detected PKS I members in UniRef

See file in PKS_I_db_and_extracts.zip

PKSDB sequences that are placed in non-PKS I branches

The following three TE sequences from PKSDB are placed in one branch that is annotated as non-PKS I branches:

- TE_megal_004_TE_001.seq_from_1_to_213
- TE_pikro_005_TE_001.seq_from_3_to_226
- TE_rifam_006_TE_001.seq_from_1_to_211

Taxonomic distribution of PKS domain sequences

See Table S2.

Comparison of the tree topologies with reference trees

The numbers of taxa and Robinson-Foulds distances of the pruned trees and of the tree pairs can be found in Table S3.

The MT domain was omitted due to its small number of taxa. As the overlap of taxa in the PP domain trees is very low the likelihood that this low distance value occurs randomly is quite hight. For the other trees the found distance between the test and reference tree is much lower than the 125750 (502 trees all-against-all minus the distance between reference tree and the analysis tree) random tree distances and represents an outlier of this (non-normal) distribution (see box plot in Figure S2).

Comparison of the tree log likelihood values

The log likelihood values of the reference trees were compared to those of the trees with metagenomic sequences and 100 trees with the same amount of taxa but random topologies. The log likelihood values of the reference trees and trees with metagenomic sequences were in all cases better and less different to each other than the log likelihood values of the random trees.

Comparison with a BLAST based method

To show that the HMM/tree based approach is more sensitive and selective than a BLAST based one we implemented a pipeline similar to SEARCHPKS. We took the same six domain sequences used there taken from Erythromocin producing PKS I (eryth_002_AT_002.seq, eryth_002_DH_001.seq, eryth_002_ER_001.seq, eryth_002_KR_002.seq, eryth_002_KS_002.seq, eryth_003_TE_001.seq) from PKSDB for searches and the same cut-off e-values. With this set up we screened the UniRef proteins and found in total 17126 domain hit sequences with the six domains. There were 2049 multi hit and 8743 single hit proteins. The annotation strings were analyzed and the proteins classified.

The single hit proteins are dominated by non-PKS I members and only a small fraction of the sequences are contributed by PKS I proteins. The PKS I to non-PKS I ratio is much higher for multi hit proteins. These results show that the BLAST based PKS search is not very selective and catches to many false positive sequences. On the other hand they

prove that combining the information of different domain searches can improve the confidence in positive PKS proteins.

Find the results in Table S4, Table S5 and Table S6.

Simulation of 454 pyrosequencing data

To test the ability of the presented pipeline to deal with short sequence that are generated by non-Sanger sequencing platforms (e.g. 454 pyrosequencing) we randomly selected 51 AT domain protein sequences found in the UniRef database and extracted subsequences of 33, 83 and 133 amino acid (representing different generations of non-Sanger sequencing). These subsequences were combined with the full UniRef database set. This sequence collection was screened with the AT domain HMM. Forty one of the sequences with 133 amino acids were found while the shorter ones were missed by the HMM (even after using a less restrictive e-value cut-off). The 133 amino acid long subsequences were aligned with 881 representative full length sequences using *hmmalign*. The same was done with the full original sequences of these subsequence. The alignments were used to create maximum likelihood trees with *PHYML*. A manual comparison of these visualized trees showed that the placement of the short sequence is in general similar to the full length proteins. This observation implies that sequences from newer non-Sanger sequencing projects with longer sequences might be successfully screened with our method while the sequence data produced by early non-Sanger projects might not offer enough information per sequence for a successful detection. Yet, found sequences can be correctly classified with maximum-likelihood trees.
