# Supplementary material for: A Computational Screen for Type I Polyketide Synthases in Metagenomics Shotgun Data
Source: PLoS One. 2008 Oct 27;3(10):e3515. doi: 10.1371/journal.pone.0003515 (PMC2568958; doi:10.1371/journal.pone.0003515)
Supplement: Supplementary file 13 [file pone.0003515.s010.doc]

	AT		DH		ER		KR		KS		MT		PP		TE		Sum for source	
	PKS	Non-PKS	PKS	Non-PKS	PKS	Non-PKS	PKS	Non-PKS	PKS	Non-PKS	PKS	Non-PKS	PKS	Non-PKS	PKS	Non-PKS	PKS	Non-PKS
	197	0	103	0	29	0	171	0	191	0	5	0	200	0	13	3	909	3
UniRef	2252	839	1290	48	642	1922	2035	225	3524	162	16	0	2727	1072	149	356	12635	4624
MFS	36	14	10	0	3	108	14	155	52	44	5	0	26	23	1	5	147	349
SGS	28	348	21	0	16	1079	42	481	69	487	2	0	35	19	1	0	214	2414
EBPRS	4	14	1	0	2	87	2	157	4	29	12	0	11	2	6	0	42	289
WLF	9	23	2	0	1	82	5	149	10	37	0	0	2	5	1	1	30	297
AMD	0	1	0	0	0	38	1	19	0	2	4	0	1	1	0	0	6	61
HGUT	0	13	0	0	0	16	0	33	0	21	1	0	1	1	0	0	2	84
Sum all source	2526	1252	1427	48	693	3332	2270	1219	3850	782	45	0	3003	1123	171	365	13985	8121
Sum for doma	3778		1475		4025		3489		4632		45		4126		536		22106	
