# Supplementary material for: A Computational Screen for Type I Polyketide Synthases in Metagenomics Shotgun Data
Source: PLoS One. 2008 Oct 27;3(10):e3515. doi: 10.1371/journal.pone.0003515 (PMC2568958; doi:10.1371/journal.pone.0003515)
Supplement: Supplementary file 14 [file pone.0003515.s011.doc]

Taxonomic group	Hit number
Actinobacteria	5642
Proteobacteria	3625
Fungi	2018
Cyanobacteria	647
Others_bacteria	459
Firmicutes	338
Animal	251
Mycetozoa	156
Alveolata	126
Viridiplantae	117
Chloroflexi	95
Planctomycetes	29
Others_eukaryotes	21
Bacteroidetes/Chlorobi	12
Others	8
