# Supplementary material for: A Computational Screen for Type I Polyketide Synthases in Metagenomics Shotgun Data
Source: PLoS One. 2008 Oct 27;3(10):e3515. doi: 10.1371/journal.pone.0003515 (PMC2568958; doi:10.1371/journal.pone.0003515)
Supplement: Supplementary file 15 [file pone.0003515.s012.doc]

	AT	DH	ER	KR	KS	PP	TE
Number of common taxa	755	501	265	1050	886	8	40
Robinson-Foulds distance	0.464096	0.544177	0.461832	0.564470	0.501699	1.000000	0.486486
