# Supplementary material for: A Computational Screen for Type I Polyketide Synthases in Metagenomics Shotgun Data
Source: PLoS One. 2008 Oct 27;3(10):e3515. doi: 10.1371/journal.pone.0003515 (PMC2568958; doi:10.1371/journal.pone.0003515)
Supplement: Supplementary file 16 [file pone.0003515.s013.doc]

AT	3216
DH	817
ER	4430
KR	1792
KS	6640
TE	231
