# Supplementary material for: A Computational Screen for Type I Polyketide Synthases in Metagenomics Shotgun Data
Source: PLoS One. 2008 Oct 27;3(10):e3515. doi: 10.1371/journal.pone.0003515 (PMC2568958; doi:10.1371/journal.pone.0003515)
Supplement: Supplementary file 17 [file pone.0003515.s014.doc]

PKS	901
Unknown	484
Acyl synthase	193
Putative PKS	188
Hypothetical protein	96
FAS	65
NRPS	62
Oxidoreductase dehydrogenase	29
Amino acid adenylation	18
Putative FAS	13
