# Supplementary material for: A Computational Screen for Type I Polyketide Synthases in Metagenomics Shotgun Data
Source: PLoS One. 2008 Oct 27;3(10):e3515. doi: 10.1371/journal.pone.0003515 (PMC2568958; doi:10.1371/journal.pone.0003515)
Supplement: Supplementary file 18 [file pone.0003515.s015.doc]

Oxidoreductase dehydrogenase	3032
Unknown	2885
Acyl synthase	1367
PKS	735
Hypothetical protein	306
Putative PKS	202
FAS	116
Thioesterase	56
NRPS	44
Hydrolase esterase	15
Amino acid adenylation	14
Putative FAS	10
Lipopeptide	1
